# Wireless Local Area Networks Threat Detection Using 1D-CNN

**DOI:** 10.3390/s23125507

**Published:** 2023-06-12

**Authors:** Marek Natkaniec, Marcin Bednarz

**Affiliations:** Institute of Telecommunications, AGH University of Science and Technology, al. Mickiewicza 30, 30-059 Krakow, Poland; mbednarz00@outlook.com

**Keywords:** MAC layer threats, network traffic analysis, threat detection, machine learning, deep learning, convolutional neural network

## Abstract

Wireless Local Area Networks (WLANs) have revolutionized modern communication by providing a user-friendly and cost-efficient solution for Internet access and network resources. However, the increasing popularity of WLANs has also led to a rise in security threats, including jamming, flooding attacks, unfair radio channel access, user disconnection from access points, and injection attacks, among others. In this paper, we propose a machine learning algorithm to detect Layer 2 threats in WLANs through network traffic analysis. Our approach uses a deep neural network to identify malicious activity patterns. We detail the dataset used, including data preparation steps, such as preprocessing and division. We demonstrate the effectiveness of our solution through series of experiments and show that it outperforms other methods in terms of precision. The proposed algorithm can be successfully applied in Wireless Intrusion Detection Systems (WIDS) to enhance the security of WLANs and protect against potential attacks.

## 1. Introduction

Wireless networks have become the most prevalent method of connecting to the global Internet. The growth in popularity of the Internet of Things (IoT) and the rapid advancement of cellular networks are the primary reasons for the wide-spread use of Wi-Fi as a connectivity technique. The IEEE 802.11 standard [1] defines the Physical (PHY) and Medium Access Control (MAC) layers for Wireless Local Area Networks (WLANs). WLANs are highly convenient for users due to their ease of use and the lack of necessary wiring. Wi-Fi is utilized in home and small office networks to provide connectivity between laptops, PCs, IoT devices, smartphones, IP cameras, and many other devices e.g., sensors. The Wi-Fi enabled sensors are designed to connect directly to a Wi-Fi network, allowing them to send data to the server without the need for a dedicated gateway device. This can make it easier to set up and manage sensor networks, as well as reducing costs and complexity. The Wi-Fi networks can also transmit sensor related data. In some cases, sensors may not have Wi-Fi connectivity built in, but can be connected to a gateway device that does. This gateway device can then transmit the sensor data over the Wi-Fi network to the server. Finally, Wi-Fi networks can be used to control and manage sensors remotely. Sensors may be programmed to respond to specific Wi-Fi frames, allowing them to be turned on or off or adjusted from a remote location [2]. This can be particularly useful in industrial or commercial settings where large numbers of sensors may need to be managed from a central server. However, due to its popularity, Wi-Fi networks have also become a target for attackers who can exploit vulnerabilities and gain access to valuable information from poorly secured networks. These attacks can have severe consequences, such as the theft of sensitive information, disruption of network services, and the compromise of user privacy. It is essential to ensure the security of wireless networks to protect against these potential threats. As newer standards for wireless networks are developed, they aim to provide increased security for users. However, the problem remains that many devices still only support older versions of the standard, creating a significant security vulnerability. Additionally, these older devices are unlikely to be replaced by newer ones due to cost and lack of awareness from users [3]. This results in a large number of users being at risk of various types of network attacks on both the physical [4] and MAC layers [5]. To address this issue, there is a need to design and develop Intrusion Detection Systems (IDSs) to identify and respond to malicious activity in wireless local area networks.

Machine learning is a powerful tool for developing Wireless Intrusion Detection Systems (WIDSs) [6]. Convolutional Neural Networks (CNNs) are one of the most popular types of deep learning algorithms which are used for this task. Wi-Fi networks are just an example of wireless networks where IDS using CNNs find their application. CNNs are also widely used for threat detection in the Internet of Vehicles (IoV) [7] and in the Internet of Things (IoT) [8] based on 5G technologies. It is one of the most effective methods of detecting threats and definitely deserves attention. By using algorithms that can learn from data and improve their performance over time, machine learning models can effectively analyze large and complex datasets to identify patterns and trends that may indicate security threats [9]. However, one of the main challenges in using machine learning for intrusion detection in wireless networks is the availability of high-quality datasets. The Aegean Wi-Fi Intrusion Dataset (AWID) [10,11] is the largest and most widely used dataset for evaluating the performance of machine learning algorithms in this context. However, the dataset has several challenges, such as class imbalance and mixed data types, that can make it difficult to use in machine learning models. Despite these challenges, the AWID dataset remains the best available option for building machine-learning models for Wi-Fi security.

The main purpose of this paper is to design, implement, and evaluate a machine learning algorithm that can detect various network attacks at the MAC layer of IEEE 802.11 wireless networks by analyzing traffic patterns. The AWID dataset is utilized to achieve this goal. This paper will elaborate on the structure of the dataset, the preprocessing steps taken, and the machine learning model employed to achieve the best classification accuracy. The ultimate outcome will be a functional algorithm that can accurately classify WLAN network traffic as normal or as a specific type of attack. The main contributions of this paper are manifold:

- a framework for threat detection in WLANs based on 1D-CNN has been developed, removing the requirement for manual selection of detection features and the creation of a model to profile normal behavior;
- a feature selection method based on the F-value metric was proposed, which indicates which feature contains the most information about the origin of the frame;
- dropout regularization technique is used to prevent overfitting during the training process, which allows to increase the randomness of parameters and ensure accurate predictions on unknown samples;
- the proposed 1D-CNN model is relatively simple, very fast, and thus consumes little energy, which was shown by the performance results;
- the model was trained on data from the complete dataset that was not considered in other works, to the best of our knowledge this is the first paper which took this into consideration, no one else has trained their model on such a large data set.

The rest of the document is organized as follows. Section 2 provides a review of previous machine learning-based threat detection research using the AWID dataset. Section 3 describes possible MAC layer attacks in IEEE 802.11 networks. The structure of the used dataset and the preprocessing steps taken to generate an input data for machine learning model are explained in Section 4. Section 5 introduces the topic of deep learning models. The concept of the proposed 1D-CNN model is shown in Section 6. Section 7 describes the training process and evaluation results. The discussion section is presented in Section 8. Finally, the conclusions and future research directions are included in Section 9.

## 2. State of the Art

This section presents a review of the current state-of-the-art in machine learning-based IDS utilizing the AWID dataset [11]. It examines various approaches and techniques that have been proposed for identifying intruders in wireless networks through machine learning and evaluates their performance using the AWID dataset. Additionally, it delves into the challenges and limitations of using machine learning for intrusion detection and highlights areas for future research. Overall, this chapter aims to provide a comprehensive understanding of the current state-of-the-art in machine learning-based IDS using the AWID dataset.

In [12] authors test several classic machine learning algorithms on a reduced set from AWID dataset both on class labeled version and attack labeled version. Various feature reduction techniques such as Information Gain and Chi-Squared statistics were used. The results showed that feature reduction can improve the accuracy, processing time, and complexity of the analysis. Their multi-class Random Tree classifier achieved 95.12% accuracy with Information Gain Attribute Evaluation chosen as a feature extraction method. Unfortunately, the authors did not take into account the interpretation of the features when selecting them. For example, in the top ten features, there were functions related to the time of sending frames when the aspect was not directly used by machine learning models.

The authors of [13] propose a deep learning model for classification. They use a window-based instance selection algorithm called “SamSelect” to balance data, and a stacked Contractive Auto-Encoder to reduce dimensionality. The resulting intrusion detection system, based on a Conditional Deep Belief Network (CDBN), can identify attack features and perform real-time intrusion detection in wireless networks. The authors achieved a detection accuracy of 97.4% with an average detection time of 1.14 ms.

The article [14] proposes a Semi-Supervised Double Deep Q-Network (SSDDQN)-based optimization method for detecting abnormal network traffic, using the Double Deep Q-Network (DDQN) as a classification algorithm, a representative of Deep Reinforcement Learning. In this method firstly the autoencoder is trained to reconstruct the dataset features properly and then a deep neural network is used as a supervised classifier. The model was tested on the NSL-KDD and AWID datasets. It achieved an accuracy of 98.99% on the latter. It is questionable how the dataset is preprocessed. The interpretation of features is omitted, which may contribute to overfitting the model relative to the dataset on which it is trained.

In [15] authors propose a basic convolutional neural network (CNN) model for a Wi-Fi intrusion detection system. The dataset is preprocessed by converting it to numerical values, normalizing it, and discarding redundant features. The authors then train the CNN model and adopt the Dropout technique to reduce overfitting risk and network training time. They also explore various network structures. Experiment results on the open AWID dataset show that the proposed algorithm has a recognition rate of up to 99.84%. Unfortunately, the authors provide too few metrics to be able to correctly compare the model and comment on its effectiveness.

Researchers in [16] deeply investigate the AWID dataset and propose an improved version of the dataset called N-AWID. They offer a few preprocessing steps which lead from the AWID dataset to N-AWID. In the beginning, every hexadecimal number is converted to decimal form, MAC addresses are converted byte by byte and the result is a 6-dimensional vector, from which the Euclidean Distance is calculated and used in the dataset. Then character data is converted to vectors using the Word2Vec algorithm and these vectors are clusterized using the KMeans algorithm. Data balancing is handled by Synthetic Minority Over-Sampling TEchnique (SMOTE) and under-sampling of the predominant normal class. After that, the dataset was evaluated on some classical machine learning models from which Random Forest scored the best accuracy of 94.22%. Still, the idea of converting MAC addresses and character data into vectors seems to be misguided. The model should not rely on this type of data because it depends on the environment in which the network is operating, so for the model to recognize threats in traffic from any network, these types of features should be omitted.

In [17], the AWID dataset was used to detect a specific class of attacks, impersonation. The authors used a Stacked Auto-Encoder to extract new features, resulting in a total of 204 features by adding 50 new features to the existing data. They then applied the C4.8 algorithm to select the best 5 features. A Support Vector Machines (SVM) model was trained and evaluated, achieving a 98.22% classification accuracy between normal behavior and impersonation attacks. One of the top five features that were selected frame.time_epoch refers to the time at which the frame was sent, but this should not be among the features, as in a real environment an attacker can send frames at different intervals, not necessarily the same as in this dataset.

Another reinforcement machine learning algorithm was used in [18]. In the AWID dataset less important features and with constant and null values were removed, categorical features were one-hot encoded resulting in 58 features in total. In this work the classic Radial Basis Function (RBF) neural network was included as a policy network in an offline reinforcement learning algorithm. The evaluation showed a 95.5% classification accuracy.

A Semi-Supervised Learning Approach was also used in [19]. This paper proposes a solution based on a ladder network. In this model firstly the supervised model with a corrupted path is fed with data and predictions are made. Then in the unsupervised phase, the same data but without labels are passed to the auto-encoder with a clear path, and unsupervised loss is calculated. After that, the general loss is calculated based on supervised training cost and unsupervised reconstruction loss. The model was trained and evaluation showed at most 99.77% accuracy in classification. A questionable aspect of set preparation is the replacement of “?” for 0. Zero implies that the field in question just carries the value zero, when in fact it is not available. In our opinion, such fields should be replaced with, for example, a negative value, because such a value cannot be present in the field in the frame, so it will mean that this field does not exist in the frame.

In [20], the authors evaluated classical machine learning models on the AWID dataset. The models tested were Extra Trees, Random Forests, and Bagging. In the end, the prediction was made using majority voting. Researchers tested these algorithms stand-alone and with majority voting at the end, and the best result was obtained with the second option with an accuracy of 96.32%.

A completely different approach was chosen in [21]. The authors present a convolutional neural network for image processing called V-CNN. The whole methodology consists of interpreting the dataset as a bunch of RGB images. Then the V-CNN model is fed with these images and trained. The evaluation shows a recall of around 99.9% in multi-class classification. However, the use of two-dimensional convolutional neural networks is much more computationally demanding, so the model may lag behind one-dimensional CNNs or classical machine learning models in terms of performance.

The researchers in [22] concentrated mainly on finding the most effective number of extracted features from the AWID dataset. They created four feature sets with 32, 10, 7, and 5 features respectively. Seven machine learning models were then trained and evaluated. The results showed that, with Cross-Validation Approach, the Random Forest achieved 99.99% accuracy using a feature set of just 7 features. However, the authors didn’t consider the correlation between the selected features and the environment where the dataset was created. Features like device MAC addresses and Wi-Fi network names are directly correlated to the laboratory where the experiment was conducted, so models trained using these features may not perform well when tested on data from outside the dataset.

In [23] researchers apply several ensemble learning methods such as Bagging, Random Forest, Extra-Trees, and XGBoost on the AWID dataset. Ensemble models leverage multiple ML algorithms to achieve higher classification accuracy than a single model. In studies, Random Forest achieved the top accuracy of 99.096% in multi-class classification, while Extra-trees algorithm closely followed with 99.017% accuracy. However, the current and previous approaches in this document have a drawback of considering laboratory-related features, potentially affecting the classification of records from other datasets.

An interesting approach is presented in [24] where the authors have shown that the chosen characteristics can potentially be applied to various IEEE 802.11 datasets, even those related to different versions of the standard. Their demonstration revealed that training an IEEE 802.11 IDS with only quartet of high importance features can result in significant effectiveness. They also demonstrated how a ML model can be improved and generalized. The conducted experiments shown that it is possible to transfer the same features from one dataset (AWID2) to another (AWID3) [25,26].

In [27] the authors proposed an approach that integrates the benefits of both feature selection and ensemble classifier, with the goal of achieving an IDS that is both efficient and accurate. In order to enhance the classification accuracy of unbalanced datasets with multiple classes, they presented an ensemble strategy that merges outputs from several classifiers (C4.5, RF, and Forest PA) using a voting classifier based on the average of probabilities combination rule. Experimental results shown that the proposed solution surpasses other methods in terms of Accuracy, F-Measure, and ADR metrics.

An approach with a hybrid deep learning model was proposed in [28]. The model consists of long short-term memory (LSTM) and CNN models. It was trained on an AWID dataset and its customized version. The customization relied on generating additional data using Time Series Generative Adversarial Network (TGAN) and merging it with an original dataset. Both models were tested against one dataset which was collected from real-time attacks in the laboratory. The model trained on the AWID+GAN dataset outperformed that which was trained on a bare AWID by 93.53% vs 84.29% in terms of accuracy.

A detection algorithm for the industrial internet of things (IIoT) based on deep learning was proposed in [29]. The authors used attention-based convolutional long short-term memory (Conv-LSTM) and bidirectional long short-term memory (Bi-LSTM) layers as feature extractors which later were used during training and testing the fully connected layers. The whole model was trained and tested on two datasets AWID and CTU-13 [30] and obtained 98.02% and 95.98% of accuracy respectively.

The papers reviewed in this study describe various approaches and techniques that have been proposed for detecting intrusions in wireless networks using machine learning, and evaluate their performance using the AWID dataset. The findings are summarized in Table 1, which presents a comparative analysis of the works examined in this study. These approaches include classic machine learning algorithms such as Random Trees and Support Vector Machines, as well as deep learning techniques such as stacked Contractive Auto-Encoders and Convolutional Neural Networks. Overall, the performance of these models has been found to be effective at detecting intrusions in the AWID dataset. However, using machine learning for intrusion detection in wireless networks also presents certain challenges and limitations. These include the need for careful preprocessing and balancing of the dataset, as well as the difficulty of ensuring the generalizability of the model to other datasets and scenarios.

To address these challenges and limitations, we propose a new approach that utilizes a 1D-CNN model consisting of a convolutional layer interlaced with dropout layers. This approach addresses the unbalanced distribution of classes by subsampling the dominant class in the data set and excluding all features associated with the laboratory where the dataset originated, resulting in improved stability for classification.

## 3. Possible IEEE 802.11 MAC Layer Threats

WLANs are vulnerable to a variety of security threats due to their inherent characteristics, such as the broadcast nature of wireless signals and the lack of physical boundaries. Moreover, the MAC protocols used in WLANs can have vulnerabilities that can be exploited by attackers to gain unauthorized access or to disrupt network communication. Many threats result from improper network configuration, e.g., using the WEP algorithm to encrypt data. Numerous other vulnerabilities have been identified, which are the result of common programming errors present in Wi-Fi products, e.g., resulting in memory overflow of Access Points when certain types of attacks using management frames are launched. Some of the vulnerabilities are related to design flaws in the Wi-Fi standard, they impact a vast majority of devices. According to the experiments conducted, there isn’t any Wi-Fi product that is entirely immune to vulnerabilities, with each one being susceptible to at least one security flaw [31]. Additionally, it is observed that a significant proportion of WLAN devices are exposed to multiple vulnerabilities. All existing Wi-Fi security protocols, including the most recent WPA3 specification, are impacted by the identified vulnerabilities [32,33]. Regular security testing of Wi-Fi products is crucial to ensure that vulnerabilities are identified and addressed. Another way to protect against MAC layer attacks is to use the IEEE 802.11w standard, also known as management frame protection, but even it does not solve many problems [34]. Especially, the transmission of control frames poses a number of threats, including the complete blockage of the network. To mitigate these vulnerabilities, it’s important to monitor wireless networks for suspicious activity and promptly investigate any potential security breaches.

As previously mentioned most IEEE 802.11 wireless networks are vulnerable to various types of MAC layer attacks. These attacks range from simple-to-deploy denial of service attacks that make use of non-encrypted management frames, to more complex attacks that involve breaking the WPA2 pre-shared key. The authors of the AWID dataset have grouped these attacks into three classes: flooding, injection, and impersonation. The dataset contains only attacks that are performed at the MAC layer. The following are types of attacks that can be performed at the MAC layer of IEEE 802.11 wireless networks.

### 3.1. Flooding

- Deauthentication flood-it involves sending a large number of deauthentication management frames with a specific destination MAC address. This results in the connection loss of a client with that MAC address, or if the broadcast address is used, the disconnection of all clients that receive the frame. The work [34] shows that even the IEEE 802.11w extension does not completely solve the problems with this attack.
- Disassociation flood-this attack is similar to a deauthentication flood, but uses disassociation management frames instead. The result is the same, with clients losing their connection to the network.
- Block Acknowledge flood-is effective against IEEE 802.11 networks that use the Block Acknowledge feature. The attacker sends a fake ADDBA message on behalf of a real client with high sequence numbers, causing the AP to not accept frames from the STA until the sequence numbers in the ADDBA message are reached. The authors in [35] exploit this vulnerability.
- Authentication request flood-it involves sending a large number of authentication request frames, which overloads the AP and can cause it to shut down and drop the wireless network [36].
- Fake Power Saving flood-it takes advantage of the Power Saving mechanism by sending a null frame on behalf of the victim with the power saving bit set to 1. The AP starts buffering frames for that client and sends the information about it in the ATIM field in the beacon frame. The client obviously is not in a sleep state therefore he ignores all ATIM messages. This causes the AP to buffer frames for the victim and can result in dropped frames [37].
- Clear-to-Send flood-it relies on the Request-to-Send (RTS)/Clear-to-Send (CTS) mechanism and involves sending a large number of CTS frames with the duration set to its maximum value. This causes STA to wait for a transmission that never occurs, preventing other clients from accessing the medium [38].
- Request-to-Send flood-this attack is similar to a CTS flood, but involves sending a large number of RTS frames, which also prevents other clients from accessing the medium [38].
- Beacon flood-it involves sending multiple beacon frames with different SSIDs, which can cause confusion for end users attempting to connect to the correct network [39].
- Probe Request flood-this attack aims to drain resources from the AP by sending a large number of probe request frames [40].
- Probe Response flood-this attack involves flooding a victim with a large number of probe response frames after receiving a probe request frame, causing the victim to have difficulty connecting to the correct network.

### 3.2. Impersonation

- Honeypot-a wireless network created by an attacker that is designed to attract unsuspecting victims. The victim is typically unaware that they are connecting to a network created by the attacker, who then has access to all the security keys and can monitor all the traffic on the network [41].
- Evil Twin-a wireless network created by an attacker that is an exact replica of an existing network used by the victim. The attacker may attempt to lure clients from the legitimate network to their own, and then launch further attacks on the newly associated devices [42].
- Caffe Latte-a method of attacking wireless networks where direct access to the access point is not necessary. The attacker simply needs to be in proximity to a device that has already authenticated to the target network. The attacker creates an identical copy of the network and tricks the device into connecting to it, and then uses ARP Request injection to collect enough initialization vectors (IVs) to crack the WEP key.
- Hirte-an extension of the Caffe Latte attack in which ARP packets are fragmented to collect even more IVs from the connected device, making it easier to crack the WEP key.

### 3.3. Injection

- ARP Injection-this method involves injecting a fake ARP Request into the wireless network. The targeted STA, whose IP matches the request, will respond with an ARP response, thereby producing more IVs that can be used to crack the WEP key.
- Fragmentation-the attacker first performs a fake authentication with the Access Point (AP) and then receives at least one frame. Since the Logical Link Control (LLC) header has many known fields, the attacker can guess the first 8 bytes of the keystream. Then, the attacker constructs a frame with a known payload, breaks it into fragments, and sends them to the AP with the broadcast address as the destination. The AP then processes these fragments, puts them together, and sends them to all STAs. Since the content of these fragments is known, the attacker can retrieve the WEP pseudo random generation algorithm which can be later used for various injection attacks.
- Chop-Chop-this attack is based on dropping the last byte of the encrypted frame and then guessing a valid Integrity Check Value (ICV) for this truncated frame using the AP. When the attacker injects a truncated frame into the network, the AP will state whether or not the ICV is valid. Once the attacker has chosen a valid ICV, he is able to retrieve one byte of keystream.

## 4. Dataset Description

### 4.1. General Description

The AWID dataset [11], created at the University of the Aegean, aimed to capture real-world wireless network traffic while various attacks, primarily at the MAC layer, were being executed. To accomplish this, the authors set up a test Wi-Fi network with 10 normal nodes that generated regular network traffic, such as web browsing and file downloading, and occasionally joined and left the network. Additionally, there was one attacker node that carried out different types of attacks and one monitor node that recorded all the traffic in the network. This data was saved in ‘.csv’ files containing values from 154 frame fields.

The dataset was divided into two subsets: “reduced” and “full”, each of which is available in two versions: one with “class-labeled” data and another with “attacks-labeled” data. In the “attacks-labeled” version, each record is assigned to either normal traffic or a specific attack, while in the “class-labeled” version, each record is assigned to either normal traffic or a specific class of attacks. Both subsets were created from two different recording sessions and are not related to each other. Both subsets were also split into training and testing parts. Table 2 and Table 3 show the class and attack distributions for both the full and reduced sets.

After unpacking all the files, the total size of the dataset is approximately 100 GB, making the preprocessing a time-consuming task. Additionally, each of the 154 features needs to be individually analyzed. The features contain different data types such as integers, floats, hexadecimal numbers, and strings. Some of them are missing in some records because not all frames include all 154 fields present in the dataset. This complexity of preprocessing is further increased by the fact that the dataset is imbalanced. The number of records for normal traffic greatly outnumbers the records for attacks. Before using this data for any machine learning algorithm, it’s essential to ensure a similar distribution of each class.

### 4.2. Preprocessing Steps

During the preparation of the dataset for use in machine learning algorithms, the following steps were taken:

- Every column containing duplicate information or being empty was removed.
- Every column containing non-numeric values, such as SSID, was removed.
- Every column related to the test environment in which the dataset was recorded, such as MAC addresses and SSID names, were removed.
- Not all frames contain all the fields described in the dataset. In such cases, the missing values were replaced with −1, as all present fields have positive values and the absence of a field was indicated by a negative one
- The dataset was balanced. Excess of normal records was dropped at least to obtain a 1:1 ratio with the number of attack records.
- Every field was converted to a decimal floating point number.
- The 36 best features were selected using the ANOVA F-value algorithm. ANOVA, or Analysis of Variance, is a statistical technique used to compare the means of different groups. It is often used to test for significant differences between the means of two or more groups. The F-value, also known as the F-ratio, measures the ratio of the variance between groups to the variance within groups. In ANOVA, the F-value is used to determine whether the means of the groups are significantly different from each other. To select features using ANOVA-F, an F-test is performed on each feature, and the features with the highest F-values are selected (see Table 4). This approach is based on the idea that the features with the highest F-values are the ones that are most likely to be important for distinguishing between different groups.
- Dataset was scaled using MinMaxScaler which is described by the following equations
  (1) $$\begin{array}{c} X\_std=(X-X.min(axis=0))/(X.max(axis=0)-X.min(axis=0)) \end{array}$$
  (2) $$\begin{array}{c} X\_scaled=X\_std\;\times \;(max-min)+min \end{array}$$
  where min, max = feature range.
- Classes were encoded using One-Hot Encoding [43]. Each class was replaced by a vector of the same length as the number of classes, which contained only zeroes. For each class, a zero in its respective vector was replaced with a one, so that each class was assigned a unique vector. This can be seen in the following example.
  $$\begin{array}{c} \mathrm{normal}=[1.0,\;0,\;0,\;0]\\ \mathrm{flooding}=[0,\;1.0,\;0,\;0]\\ \mathrm{injection}=[0,\;0,\;1.0,\;0]\\ \mathrm{impersonation}=[0,\;0,\;0,\;1.0] \end{array}$$

**Table 4:** Chosen features names.

Feature Name	F-Value	Feature Name	F-Value
wlan.fc.pwrmgt	653,236.4	radiotap.channel.type.cck	83,735.6
radiotap.channel.type.ofdm	83,234.3	wlan.wep.icv	71,694.1
radiotap.datarate	66,488.8	wlan.fc.protected	55,439.5
wlan.duration	52,483.1	wlan.wep.key	46,696.8
wlan.fc.type_subtype	42,291.6	wlan.qos.ack	36,927.5
wlan.qos.amsdupresent	36,834.8	wlan.fc.ds	34,711.1
wlan.qos.tid	33,638.9	frame.len	25,735.4
wlan.qos.eosp	24,979.2	data.len	24,374.6
wlan.frag	22,569.9	wlan.seq	14,606.9
wlan_mgt.fixed.beacon	14,334.8	wlan.wep.iv	12,185.2
wlan.fc.retry	8792.1	wlan.fc.frag	5998.5
wlan.qos.bit4	5339.3	wlan.qos.txop_dur_req	5339.3
wlan_mgt.fixed.capabilities.cfpoll.ap	4863.4	wlan_mgt.tim.bmapctl.multicast	3528.0
wlan.tkip.extiv	3300.0	wlan_mgt.fixed.capabilities.apsd	3036.0
wlan_mgt.fixed.capabilities.radio_measurement	3035.8	wlan_mgt.fixed.capabilities.agility	3031.4
wlan_mgt.fixed.capabilities.del_blk_ack	3026.6	wlan_mgt.fixed.capabilities.pbcc	3026.1
wlan_mgt.fixed.capabilities.ibss	3025.2	wlan_mgt.fixed.capabilities.dsss_ofdm	3025.2
wlan_mgt.fixed.capabilities.imm_blk_ack	3025.1	wlan_mgt.fixed.capabilities.spec_man	3024.1

### 4.3. Dataset Division

The training set was composed of a subset of the dataset called AWID-CLS-F-Trn and a validation set from AWID-CLS-F-Tst, due to the high dominance of the normal traffic class. The large size of these sets allowed for the undersampling of the largest class without issue. The testing set was composed of AWID-CLS-R-Trn. The preprocessing steps discussed in the previous section were applied to all sets. The selected features are listed in Table 4.

## 5. Deep Learning Models

Machine learning is the process of building systems that recognize patterns in input data and generate output based on these patterns, without explicit instructions. Deep learning, a subset of machine learning, uses artificial neurons organized in layers to create models. A typical deep learning model includes an input layer, one or more hidden layers, and an output layer, with connections between consecutive layers (excluding input and output). This model is known as a neural network.

The input layer receives data from the dataset and passes it to the next layer. The hidden layers perform calculations and learning. Each neuron takes values from connected neurons in the previous layer, multiplies them by their weights, adds a bias, and sends the result to an activation function. The activation function determines whether the neuron should activate and what its output value should be. Activation functions include ReLU, sigmoid, and tanh. Learning involves adjusting the weights and biases to minimize the loss function, which measures the difference between the desired and actual output. For example, in a classification task, the loss function would describe the difference between the predicted probability distribution and the desired distribution. The loss is calculated in the last layer and then backpropagated to the preceding layers, where the neurons adjust their weights and biases based on the results.

Machine learning is a very powerful tool and is widely used in plenty of domains. The main tasks that these algorithms are capable of are: regression (e.g., predicting the cost of insurance, predicting the state of radio channel), classification (e.g., distinguishing humans from animals, malware or spam classification) [44], filtration (e.g., filtration of digital signals), and data analysis (e.g., finding patterns in data). Popular and useful deep learning models include:

- Multilayer Perceptron (MLP)-model composed of fully connected layers, e.g., used in regression, classification, dimensionality reduction
- CNN-model composed of convolutional layers, e.g., used in image processing and classification, signal filtering and analysis.
- Long Short-Term Memory (LSTM)-a deep learning model which has feedback connections allowing it to process whole sequences of data, e.g., used in handwriting recognition and speech recognition.
- Autoencoder-a deep learning model which consists of two submodels, encoder, and decoder. Its main goal is to reduce input to a lower dimension using the encoder and then reconstruct the input data from it using the decoder, e.g., used in dimensionality reduction, data compression, and decompression, signal denoising.

### 5.1. Convolutional Neural Networks

CNN is the model which allowed the use of image processing in machine learning. CNNs perform convolution on the input data, which extracts important information and passes it to the next layers for tasks such as classification. This can be thought of as applying a filter to the input data, which filters out unnecessary information. During learning, the CNN adjusts the weights of these filters based on loss function value to extract only what is needed for a given task. Pooling layers can also be placed between convolutional layers to reduce the dimensionality of the input data by taking the maximum or average value of several pixels or samples and mapping it to one feature. When we talk about image processing and CNNs, we are referring to two-dimensional Convolutional Neural Networks (2D-CNNs). However, CNNs can also be used for other applications besides image processing. For example, one-dimensional Convolutional Neural Networks (1D-CNNs) can be used for classification tasks [45,46]. These can be thought of as a one-dimensional digital signal filtering, since the data is a set of 1D vectors consisting of IEEE 802.11 frame fields. In this case, the convolution is performed by kernel sliding only in one dimension. We can define its length, stride that is the number of elements to shift over, the number of filters (distinct kernels), and padding (if the kernel does not fit into input data).

Through the process of parameter learning, the convolutional layers within the CNN progressively acquire the ability to identify features at different levels of complexity. Initially, they detect elementary aspects such as edges and corners, gradually advancing to more intricate characteristics like shapes and textures. This hierarchical learning mechanism empowers CNNs to develop a structured understanding of the data they encounter. This capability makes CNNs exceptionally well-suited for a diverse range of tasks, including image recognition, object detection, and notably, intrusion detection systems in wireless networks. In the field of threat detection, CNNs excel at analyzing network traffic and detecting attack patterns that may be hidden in wireless communications. By analyzing data packets and identifying anomalies or malicious activities, CNN-based threat detection systems play an important role in protecting wireless networks from various security threats.

### 5.2. Regularization

One of the common problems with neural networks is overfitting, which occurs when the model has learned patterns that are specific to the training dataset, but not generalizable to other datasets. This leads to poor performance on a test dataset. There are several methods to prevent overfitting, including L1 and L2 regularizations. These methods add a penalty term to the loss function, which results in smaller weights in the model’s neurons and a simpler model.

**Figure 1:**
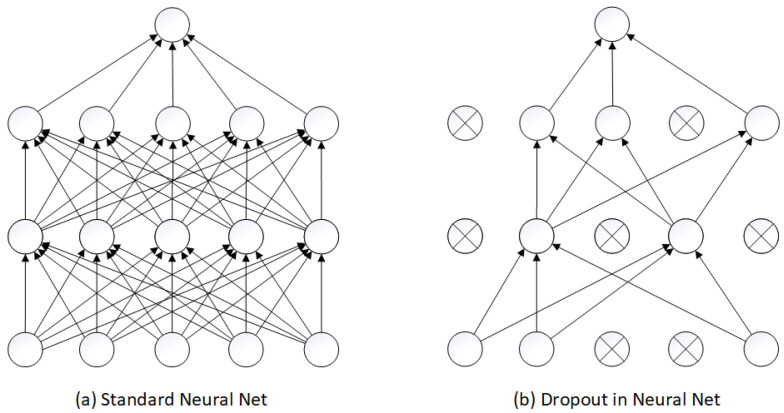
An example of applying dropout to a neural network.

The most popular regularization method is dropout [47]. The dropout layer randomly “turns off” some neurons, temporarily disabling their backward and forward connections, effectively creating a new model from the base model. Every neuron in the dropout layer has a probability of turning off by p. For example, if we have a dropout layer with 100 neurons, the probability of turning off a neuron (also known as the dropout rate) is set at 0.2. This means that in each iteration, 20 neurons will be randomly selected and turned off, temporarily disabling their backward and forward connections. That mechanism is presented in Figure 1.

## 6. Proposed 1D-CNN Model

Our motivation for choosing CNN model is its ability to run efficiently without requiring significant computational power during regular operation. Although training the CNN model itself is very time-consuming process and requires substantial computational resources, once we have a trained model, we can easily use it on client devices that require lightweight solutions. Our model finds a valuable application in the IoT domain, where numerous devices operate with limited computational and power capabilities. Therefore, low energy consumption during the operation of our model is crucial for our threat detection system, as we demonstrate in the further part of our work.

**Figure 2:**
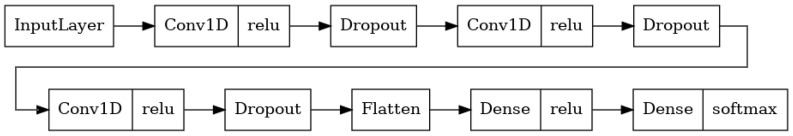
The proposed deep learning 1D-CNN model.

After preprocessing, our records are represented as one-dimensional series of float data type values, so we will use the one-dimensional version of convolutional layers. Following the series of convolutional layers, the next layer is a fully connected layer. Each of these layers is activated by a ReLu function. Dropout layers will be placed between the convolutional layers to prevent overfitting and ensure accurate predictions on unknown samples. Additionally, an L2 kernel regularizer will be applied to the first dense layer, as it has the most trainable parameters, to further prevent overfitting of the model. The last layer is a dense layer with 4 neurons in the case of multi-class classification and with 2 neurons in the case of binary classification activated by softmax function. The proposed machine-learning model is presented in Figure 2.

The softmax function normalizes the output of the layer by calculating the probability distribution among all classes. The loss function used in this model is the Categorical Cross-Entropy. This loss function is often used in classification tasks, where the goal is to predict the class label of an input sample. In a classification task with n classes, the categorical cross-entropy loss is defined as $loss=-\sum y_{i}\;\times \;\log y_{pred\_i}$ where $y_{i}$ is the true label (a one-hot encoded vector of length n) and $y_{pred}$ is the predicted probability distribution over the n classes. The loss is a scalar value that measures the difference between the true label and the predicted probabilities. The specific parameters of each layer are included in Table 5.

## 7. Model Training and Evaluation

### 7.1. Environment Overview

The whole preprocessing of data and training scripts were written in Python programming language using Tensorflow Keras API v2.9.2. The training and evaluation environment was on Google Colab Pro using a Tesla T4 GPU and 24 GB of RAM memory. Additionally, both models were tested locally on HP Envy 15 x360 laptop with Ryzen 7 4700 U and 32 GB of RAM memory and an integrated graphics card. The specific information about the operating system, programming language, and libraries is listed below.

- Ubuntu 22.04.2 LTS x86_64
- Python 3.9.12
- numpy 1.20.3
- pandas 1.3.4
- scikit_learn 1.2.1
- tensorflow 2.11.0
- wandb 0.13.10

**Figure 3:**
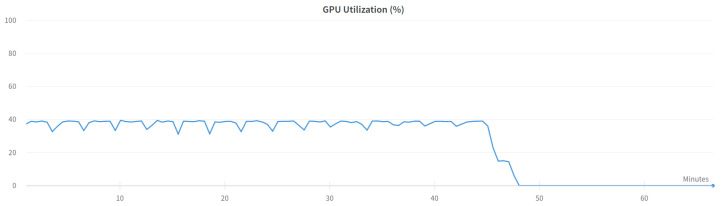
GPU utilization for binary model.

**Figure 4:**
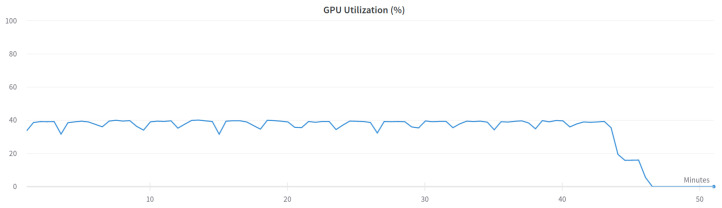
GPU utilization for multi-class model.

**Figure 5:**
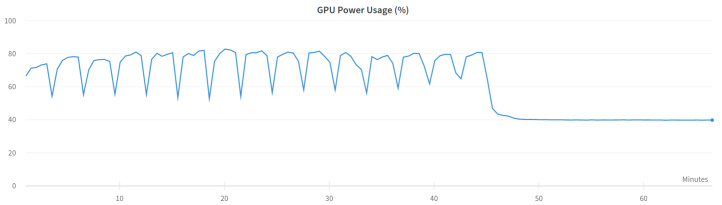
GPU power usage for binary model.

**Figure 6:**
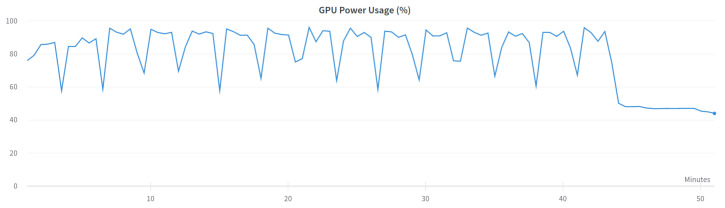
GPU power usage for multi-class model.

Two versions of the classification model were trained-binary and multi-class classification. In the binary model, only the prediction of whether the input frame is from an attack or not is made. In the multi-class classification model, the prediction of which class the input frame belongs to-normal, injection, impersonation, or flooding is made. The binary model was trained using the following settings presented in Table 6 and the multi-class model was trained using settings in Table 7. Figure 3 and Figure 4 show the GPU utilization during training of the models. We can see that GPU is not fully utilized. The main reason behind this is the fact that both models were trained on the Google Colab platform which does not grant us direct access to the hardware. The components might be shared among other users and a single user cannot control the GPU occupation for his own purposes. Figure 5 and Figure 6 show the power usage of GPU during model training. The power usage varies during training but the Nvidia Tesla T4 GPU can use at most 70 W of power which makes it very energy efficient. The proposed 1D-CNN models are available for validation on the Github repository [48].

### 7.2. Obtained Results

The following metrics were calculated to measure overall model performance:

- Precision = TP/(TP+FP)
- Recall = TP/(TP+FN)
- F1 score = 2×(Recall×Precision)/(Recall+Precision)
- AUC = Area under ROC (receiver operating characteristic curve) curve

Where TP means true positive, e.g., in a binary classification where the model predicted the attack and the real label of the sample is an attack. FP means false positive, e.g., in a binary classification where the model predicted the attack and the real label of the sample is normal traffic. FN means false negative, e.g., in a binary classification where the model predicted normal traffic and the real label of the sample is an attack.

Precision measures the proportion of true positive predictions made by the model, while recall measures the proportion of actual positive samples that were correctly identified by the model. The recall is very important if there cannot be any false negatives, e.g., because it is very expensive for us. F1-score is a metric that combines precision and recall, and is useful when a balance between the two is desired. It is particularly useful when the dataset has a large number of negatives. AUC tells us about classifiers’ ability to distinguish between classes. After training each model the following results were obtained (see Table 8 and Table 9).

**Figure 7:**
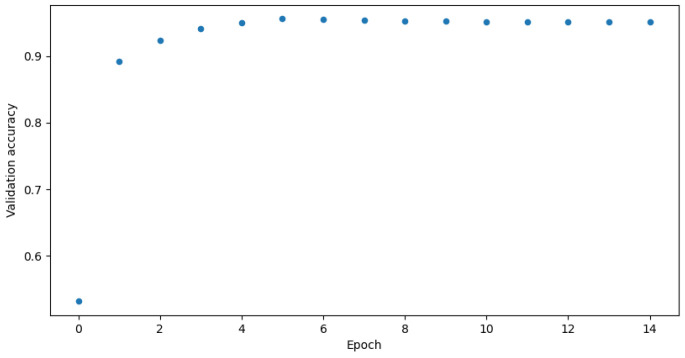
Validation accuracy for binary model.

**Figure 8:**
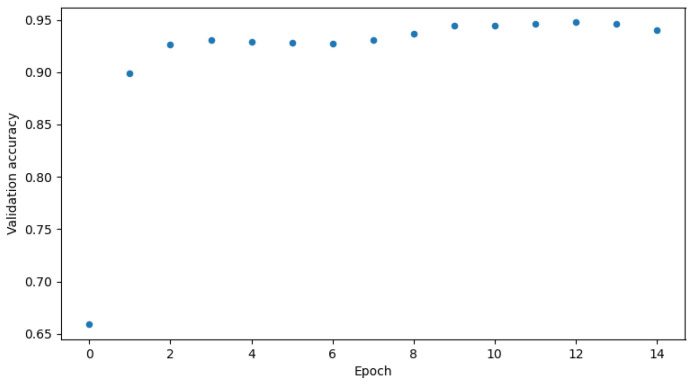
Validation accuracy for multi-class model.

**Figure 9:**
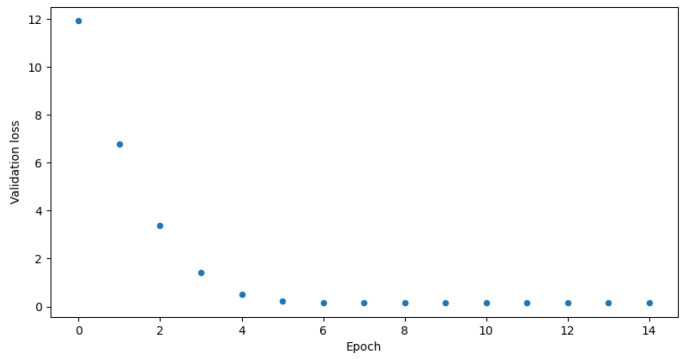
Validation loss for binary model.

**Figure 10:**
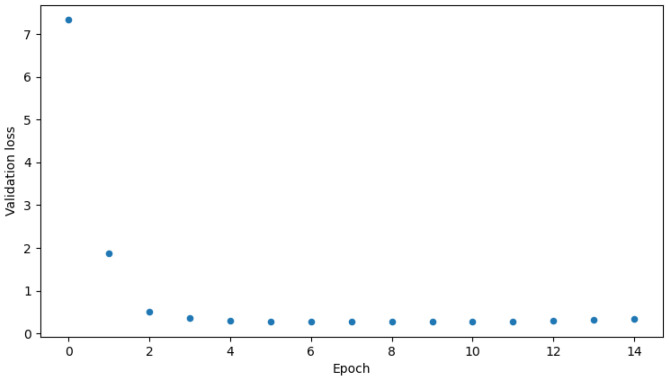
Validation loss for multi-class model.

The proposed algorithm reaches great accuracy which is over 90% in both binary and multi-class classification. It is 95.2% in the case of binary classification and 94.0% in the case of multi-class. Figure 7 and Figure 8 show validation accuracy for both binary and multi-class models respectively over each epoch of training. Figure 9 and Figure 10 show validation loss values over each epoch for both models.

The biggest issue was the overfitting phenomenon. If one looks at the Table 6 and Table 7 a very low learning rate can be seen. Adjusting it was crucial for proper model training. Other solutions like dropout layers and kernel regularizers, were described in the preceding sections and were also really helpful. They slowed down the learning process a bit, but made the accuracy charts in Figure 7 and Figure 8 smoother. As seen in Figure 9 and Figure 10, the models came close to overfitting, but ultimately this did not happen, and regularization methods proved to be effective. The validation loss remained unchanged in the last few epochs. The same applies to the Figure 7 and Figure 8 where validation accuracy is presented for binary and multi-class models, respectively. In the first case, the validation accuracy remained constant in the final few epochs, whereas in the case of multi-class classification, it slightly decreased in the last epoch.

**Figure 11:**
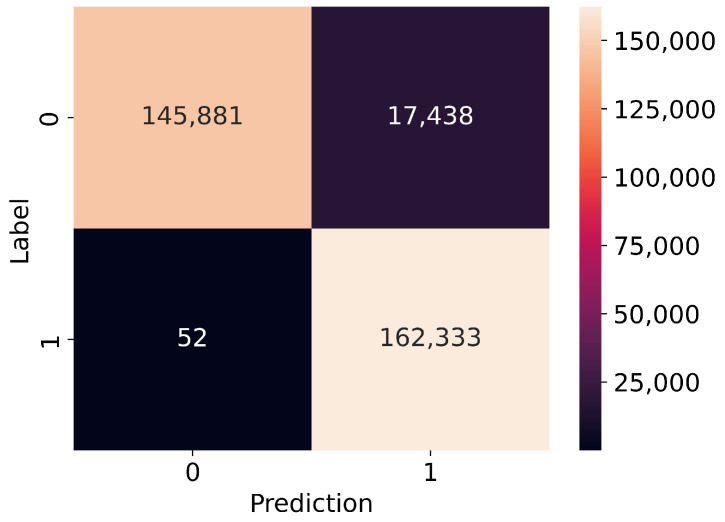
Confusion matrix for binary model: 0-normal, 1-attack.

**Figure 12:**
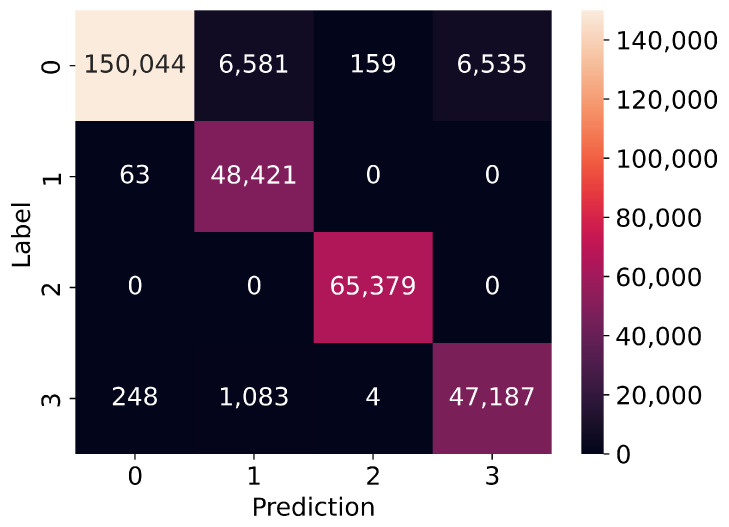
Confusion matrix for multi-class model: 0-normal, 1-flooding, 2-injection, 3-impersonation.

The most interesting part is in Figure 11 and Figure 12 which present confusion matrices for binary and multi-class models. It is clear that our model has a slight bias towards predicting positives over negatives. It is worth noting that the multi-class model has exceptional performance in distinguishing different positive classes, particularly in the injection and flooding classes. That might be because of the specific format of the frames that were injected during these attacks, e.g., unusual very small fragmented frames in a fragmentation attack or specific flooding frames, e.g., deauthentication or disassociation to broadcast. However, our convolutional neural network is weaker in predicting frames from the impersonation class, as it is difficult to distinguish these attacks from real access points operation. Additionally, some frames used in impersonation attacks are also used in flooding attacks, leading to false positives in the normal and flooding classes.

Table 10 and Table 11 present the number of records of each class and how many of them have been predicted correctly. Both models handle very well samples from attacks. The binary model predicted correctly 99.97% of frames from attacks. The multi-class model predicted correctly 99.87% of flooding class samples and 97.25% of impersonation class samples. What is worth mentioning is that every sample from the injection class was predicted correctly.

Both models were able to perform a prediction of 325,704 samples in only 5.81 s using a batch size of 300 samples. When the batch size was raised to 1000 samples the prediction took 4.51 s which makes it 56,509.20 samples per second in the first case and 72,218.18 samples per second. This was achieved using only a Ryzen 7 4700 U CPU. The result will be even better when using a dedicated GPU. Moreover, during the test, the CPU consumed only 25 W of power which makes it a very efficient solution in terms of energy efficiency. It is also worth mentioning that our model does not affect network traffic in any way, so we do not introduce any latency in communication.

## 8. Discussion

The comparison between other state-of-the-art models is presented in Table 12. Our models achieved precision scores of 0.946 and 0.972 for binary and multi-class classification, respectively. This indicates that our approach has higher precision than any other model in Table 12 that reported this metric. The multi-class model has the highest precision, indicating that this configuration provides more relevant results and fewer irrelevant ones than any other algorithm in this comparison. In terms of accuracy, our approach is comparable to some classical machine learning models like Random Forest from [12] and one of the deep learning models such as DRL+RBFNN from [18]. It should be noted that most state-of-the-art approaches have higher accuracy than our models. This could be attributed to the imbalance in the testing sets used. Only SamSelect+SCAE+CDBN [13] and Support Vector Machines [17] addressed this issue by balancing their testing datasets. Other approaches that outperformed our models in terms of accuracy did not balance their testing datasets, which led to higher accuracy scores, as one class made up a majority of the data set.

In our approach, the binary and multi-class classifications scored a recall value of 0.951 and 0.932, respectively. However, all other models in comparison that reported this metric in Table 12 scored higher values. Only the binary model came close to the DRL+RBFNN from [18] which scored 0.955. A lower recall measure means that our CNN deep learning models return less relevant results than other reference algorithms. Comparing F1 scores is a common way to evaluate multiple machine learning models. The F1-score combines the precision and recall of a classifier into a single metric by taking the harmonic mean of both and therefore it should be used for comparison of multiple models. Thus, as shown in the previous section, it consumes minimal amounts of energy. Upon analyzing Table 12, it can be seen that our two models, both binary and multi-class, perform slightly worse than the SamSelect+SCAE+CDBN deep learning model from [13] and the Support Vector Machines from [17] when it comes to F1-score. Despite that, it is worth mentioning that our models are surely less complex than these solutions which is definitely an advantage considering the slightly worse F1-score. However, our approach is more effective compared to the Double Deep Q-Network [14], CNN [15], DRL+RBFNN [18] and Random Forest [23] models. It should be noted that these papers did not take into account the dataset imbalance which is crucial for building a reliable and balanced model. Our multi-class model achieves a better F1-score, indicating that our model is more balanced and reliable in predicting all classes. Considering AUC, only two papers provided this metric, nevertheless proposed approach achieves the best results with the multi-class model on top with a 0.99 AUC score and a binary model with a 0.972 score while Random Forest [12] and SamSelect+SCAE+CDBN [13] scored 0.704 and 0.978 respectively. This means that the multi-class model is the best in distinguishing different classes.

In order to increase the effectiveness of the model, the set was balanced in terms of class distribution. This is one solution. It was also possible to introduce weights as to the prediction that would emphasize the detection of threats. Nevertheless, the model was tested on real traffic and proved to be effective. Moreover, the model does not have to work directly on the first line of threat detection and can, for example, be used to finally classify whether a suspicious frame really comes from a network attack.

The proposed machine learning model analyzes only one frame at a time by which to predict its origin it can only analyze its fields. The whole process of prediction is to catch the differences in the fields of frames, between normal traffic and that coming from attacks. The model has no knowledge of what frames appeared before, or at what time interval, which is why the prediction must work this way. If an attacker modifies the wlan_mgt.fixed.timestamp field it will not affect the machine learning model because it is not taken into account during prediction. If the attacker modifies eg. the following fields:

- wlan_mgt.fixed.timestamp,
- wlan_mgt.fixed.capabilities.imm_blk_ack,
- wlan_mgt.fixed.capabilities.del_blk_ack.

There is a high probability that the proposed machine learning model will classify the given frame as coming from an attack.

We decided to use 1D-CNN because of the nature of our dataset. 2D-CNNs as input, receive a two-dimensional array of numerical data, which can be, for example, an image or some kind of data matrix. In our dataset, a single record is a one-dimensional array with field values from the IEEE 802.11 frame. Therefore, we decided to choose 1D-CNN which as input receive data in just such a format. This explains that there is no issue with input data format for machine learning model.

The biggest challenge in building the proposed model was the overfitting phenomenon, which was addressed by adjusting the learning rate and using regularization techniques such as dropout layers and kernel regularizers. Despite these measures, the model was still close to overfitting, as shown in the confusion matrices for binary and multi-class classification. These matrices showed that the model was slightly biased toward predicting positives rather than negatives, and had strong performance in distinguishing different positive classes, particularly injection and flooding attacks. However, it was weaker in predicting frames from the impersonation class, which are difficult to distinguish from real access points operation.

Throughout the model building process, we conducted extensive training iterations, enabling us to fine-tune the hyperparameters. Additionally, we created a hyperparameter tuning script using Tensorflow Keras Tuner. Regrettably, this approach did not yield superior results, leading us to conclude that manually adjusting the hyperparameters proved to be entirely satisfactory. Nonetheless, exploring more advanced methods for hyperparameter selection may be the subject of future research in this area.

## 9. Conclusions

In this paper, we propose a deep learning model that uses a 1D-CNN to identify malicious activity patterns in WLANs traffic. We also define a preprocessing methodology for using its results in CNN model. We propose a feasible solution for feature extraction and machine learning algorithm that performs very well on testing data. It is noteworthy to mention that our model was trained on data from the complete AWID dataset. The proposed model demonstrates exceptional performance with an accuracy rate of over 95%, rivaling the performance of state-of-the-art models and surpassing some traditional machine learning models. The proposed deep learning model effectively processes the unbalanced and diverse data types in the AWID dataset to achieve high accuracy in classification for Wi-Fi security.

In a direct comparison with other models, our model demonstrated unparalleled performance in precision and F1-score, showcasing its robustness and excellence in these crucial metrics. Although its recall values were lower than other algorithms, the binary model’s recall score was comparable to that of the DRL+RBFNN model. The proposed model offers a promising solution for addressing the challenges of wireless network security, and its outstanding performance in precision, recall, and F1-score makes it suitable for use in WIDS or as a foundation for developing more advanced machine learning algorithms. Overall, the paper illustrates the effectiveness of utilizing deep learning techniques for addressing the challenges of wireless network security and highlights the potential of the proposed model to advance the state of the art in this field.

Despite the widespread use of Wi-Fi networks, many of them still operate on older, vulnerable standard extensions. Additionally, new equipment that supports the latest amendments can be cost-intensive. As such, there is a significant need for further developments in algorithms for threat detection in WLANs. Future work on the proposed algorithm could include exploring the use of a stacked auto-encoder to extract relevant features from the dataset, or utilizing alternative machine learning models such as LSTM to take advantage of the sequential nature of the data and timestamps included in it. Unlike traditional neural networks, LSTMs possess feedback connections that allow them to process entire sequences of data, not just individual records. Another approach to consider is utilizing the unbalanced class distribution in the dataset. There is also a possibility to train a machine learning model that focuses on detecting anomalies rather than classifying frames. This approach may prove particularly effective due to the large amount of normal traffic present in the dataset. Moreover, our model uses fields from IEEE 802.11 frames as features which allows for testing it on the other datasets, e.g., AWID3 [25].

## Figures and Tables

**Table 1:** Comparison of ML models enabling detection of threats in WLANs based on AWID dataset.

Paper	Model(s)	Dataset(s)	Detection of MAC Layer Threats			Features	Features Selection Method	Limitations	Year
			Flooding	Impersonation	Injection				
[12]	OneR, J48, RF ^1^, RT ^2^, AB ^3^	AWID2	+	+	+	9	Information Gain and Chi-Squared statistics	Interpretation of the features is missing	2016
[20]	Bagging, RF, Extra Trees	AWID2	+	+	+	20	Extra Trees	Only few metrics are considered	2016
[21]	V-CNN	AWID2	+	+	+	71	N/A	Only few metrics are considered	2018
[22]	OneR, J48, RF, NB ^4^, Bagging, Simple Logistic, ML Perceptron CS	AWID2	only as Attack	only as Attack	only as Attack	7	Correlation Feature Selection with Best First Search	The laboratory-related features are considered	2018
[23]	Bagging, RF, Extra Trees, XGBoost	AWID2	+	+	+	18	Manual	The laboratory-related features are considered	2018
[19]	Semi-Supervised Learning	AWID2	+	+	+	95	N/A	Datase preparation phase is questionable	2019
[13]	CDBN ^5^	AWID2, LITNET	+	−	+	20	Stacked Contractive Auto-Encoder	The laboratory-related features are considered.	2020
[15]	CNN ^6^	AWID2	+	+	+	45	Manual	Only few metrics are considered	2020
[17]	C4.8	AWID2	−	+	−	5	Stacked Auto-Encoder	One of the top features is based on frame transmission time	2020
[27]	C4.5, RF, Forest PA	NSL-KDD, AWID2, CIC-IDS2017	+	+	+	8	Correlation-based feature selection Bat algorithm	One of the top features is based on frame transmission time	2020
[14]	DDQN ^7^	AWID2, NSL-KDD	+	+	+	49	N/A	Interpretation of the features is missing	2021
[18]	RBFNN ^8^, DRL ^9^+RBFNN	AWID2, NSL-KDD, UNSW-NB15, CICIDS2017, CICDDOS2019	+	+	+	58	Manual	High complexity of deep learning models	2021
[24]	Light GBM ^10^	AWID2, AWID3	+	+	+	16	Stacked Auto-Encoder	Unidentified	2022
[28]	LSTM ^11^, CNN	AWID2	only as Attack	only as Attack	only as Attack	12	SHapley Additive exPlanations	One of the top features is based on frame transmission time	2022
[29]	Conv-LSTM, Bi-LSTM	AWID2, CTU-13	+	+	+	50	N/A	Dataset preprocessing description is missing	2022
Proposed model	1D-CNN	AWID2	+	+	+	36	ANOVA F-value	Unidentified	2023

^1^ Random Forest. ^2^ Random Trees. ^3^ AdaBoost. ^4^ Naive Bayes. ^5^ Convolutional Deep Belief Networks. ^6^ Convolutional Neural. ^7^ Double Deep Q-Network. ^8^ Radial Basis Function Neural Networks. ^9^ Deep Reinforcement Learning. ^10^ Gradient Boosting Machine. ^11^ Long Short-Term Memory.

**Table 2:** Class distribution and attack distribution in full set [11].

Full Set							
**AWID-ATK-F-Trn**		**AWID-ATK-F-Tst**		**AWID-CLS-F-Trn**		**AWID-CLS-F-Tst**	
amok	12,416	amok	3856	flooding	1,211,459	flooding	197,933
arp	1,529,284	arp	500,823	impersonation	1,884,378	impersonation	477,514
auth_request	93,011	auth_request	34,833	injection	1,530,373	injection	523,942
beacon	170,826	beacon	5498	normal	157,749,037	normal	47,325,477
cafe_latte	1,860,780	cafe_latte	16,719				
deauthentication	817,954	chop_chop	22,879				
evil_twin	23,598	cts	38,359				
fragmentation	1098	deauthentication	33,870				
normal	157,749,037	disassociation	34,871				
probe_response	117,252	evil_twin	27,045				
		fragmentation	240				
		hirte	433,750				
		normal	47,325,477				
		power_saving	13,551				
		probe_request	10,981				
		probe_response	8578				
		rts	13,536				

**Table 3:** Class distribution and attack distribution in reducet set [11].

Reduced Set							
**AWID-ATK-R-Trn**		**AWID-ATK-R-Tst**		**AWID-CLS-R-Trn**		**AWID-CLS-R-Tst**	
amok	31,180	amok	477	flooding	48,484	flooding	8097
arp	64,608	arp	13,644	impersonation	48,522	impersonation	20,079
authentication_request	3500	beacon	599	injection	65,379	injection	16,682
beacon	1799	cafe_latte	379	normal	1,633,190	normal	530,785
cafe_latte	45,889	chop_chop	2871				
deauthentication	10,447	cts	1759				
evil_twin	2633	deauthentication	4445				
fragmentation	770	disassociation	84				
normal	1,633,190	evil_twin	611				
probe_response	1558	fragmentation	167				
		hirte	19,089				
		normal	530,785				
		power_saving	165				
		probe_request	369				
		rts	199				

**Table 5:** Detailed model architecture.

Layers
Input Layer
Convolutional 1D (num_of_filters = 128, kernel_size = 1, strides = 1, activation = ReLU, padding = same)
Dropout (dropout_probability = 0.5)
Convolutional 1D (num_of_filters = 64, kernel_size = 1, strides = 1, activation = ReLU, padding = same)
Dropout (dropout_probability = 0.5)
Convolutional 1D (num_of_filters = 32, kernel_size = 1, strides = 1, activation = ReLU, padding = same)
Dropout (dropout_probability = 0.5)
Flatten
Dense (units = 100, activation = ReLU, kernel_regularizer = L2 (factor = 0.1)
Output Layer (activation = softmax)

**Table 6:** Settings for binary model.

Optimizer	Adam
Learning rate	10^−7^
Batch size	50
Number of epochs	15

**Table 7:** Settings for multi-class model.

Optimizer	Adam
Learning rate	10^−6^
Batch size	50
Number of epochs	15

**Table 8:** Results for binary model.

Metric	Value
Accuracy	0.946
Precision	0.946
Recall	0.951
F1-score	0.946
AUC	0.972

**Table 9:** Results for multi-class model.

Metric	Value
Accuracy	0.955
Precision	0.972
Recall	0.934
F1-score	0.951
AUC	0.99

**Table 10:** Class prediction score for binary model.

Class	Number of Records	Predicted Records	% Predicted
Normal	163,319	145,881	89.32%
Attack	162,385	162,333	99.96%

**Table 11:** Class prediction score for multi-class model.

Class	Number of Records	Predicted Records	% Predicted
Normal	163,319	150,044	91.87%
Flooding	48,484	48,421	99.87%
Injection	65,379	65,379	100%
Impersonation	48,522	47,187	97.25%

**Table 12:** Comparison with other state of the art models.

Model	Accuracy	Precision	Recall	F1-Score	AUC
1D-CNN-binary (proposed)	0.946	0.946	0.951	0.946	0.972
1D-CNN-multi-class (proposed)	0.955	0.972	0.934	0.951	0.99
Random Forest [12]	0.9512	0.91	-	-	0.704
SamSelect+SCAE+CDBN [13]	0.974	-	0.976	0.971	0.978
Double Deep Q-Network [14]	0.9899	0.9699	-	0.9325	-
CNN [15]	0.9984	-	-	-	-
Support Vector Machines (SVM) [17]	0.9822	-	0.9764	0.9821	-
DRL+RBFNN [18]	0.955	0.914	0.955	0.934	-
Random Forest [23]	0.99096	0.96	0.96	0.95	-

## Data Availability

The data presented in this study are available on request from the corresponding author.

## Abbreviations

The following abbreviations are used in this manuscript:

1D-CNN	one-dimensional Convolutional Neural Network
2D-CNN	two-dimensional Convolutional Neural Network
AP	Access Point
ARP	Address Resolution Protocol
ATIM	Announcement Traffic Indication Message
AWID	Aegean Wi-Fi Intrusion Dataset
CAE	Contractive Auto-Encoder
CDBN	Conditional Deep Belief Network
CNN	Convolutional Neural Networks
CTS	Clear to Send
DDQN	Double Deep Q-Network
DRL	Deep Reinforcement Learning
FN	False Negative
FP	False Positive
GPU	Graphics Processing Unit
ICV	Integrity Check Value
IDSs	Intrusion Detection Systems
IoT	Internet of Things
IV	Initialization Vector
IP	Internet Protocol
LLC	Logical Link Control
LSTM	Long Short-Term Memory
MAC	Medium Access Control
MLP	Multilayer Perceptron
PHY	Physical
RBF	Radial Basis Function
RBFNN	Radial Basis Function Neural Network
RF	Random Forest
RTS	Request to Send
SMOTE	Synthetic Minority Over-Sampling TEchnique
SSID	Service Set Identifier
SSDDQN	Semi-Supervised Double Deep Q-Network
SVM	Support Vector Machine
TP	True Positive
WEP	Wired Equivalent Privacy
WLANs	Wireless Local Area Networks
WPA	Wi-Fi Protected Access
